# Characterization of retinal function and structure in the MPTP murine model of Parkinson’s disease

**DOI:** 10.1038/s41598-022-11495-z

**Published:** 2022-05-09

**Authors:** Katie K. N. Tran, Vickie H. Y. Wong, Jeremiah K. H. Lim, Ali Shahandeh, Anh Hoang, David I. Finkelstein, Bang V. Bui, Christine T. O. Nguyen

**Affiliations:** 1 Ocular Biomarkers Laboratory, Department of Optometry and Vision Sciences, University of Melbourne, Parkville, VIC 3010 Australia; 2 Parkinson’s Disease Laboratory, The Florey Institute of Neuroscience and Mental Health, The University of Melbourne, Parkville, VIC 3010 Australia; 3 Present Address: Caring Futures Institute, College of Nursing and Health Sciences, Flinders University, Bedford Park, SA 5042 Australia

**Keywords:** Retina, Biomarkers

## Abstract

In addition to well characterized motor symptoms, visual disturbances are increasingly recognized as an early manifestation in Parkinson’s disease (PD). A better understanding of the mechanisms underlying these changes would facilitate the development of vision tests which can be used as preclinical biomarkers to support the development of novel therapeutics for PD. This study aims to characterize the retinal phenotype of a mouse model of dopaminergic dysfunction and to examine whether these changes are reversible with levodopa treatment. We use a 1-methyl-4-phenyl-1,2,3,6-tetrahydropyridine (MPTP) mouse model of PD to characterize the neurotoxic effects of MPTP on in vivo retinal function (electroretinography, ERG), retinal structure (optical coherence tomography, OCT) and retinal dopaminergic cell number (tyrosine hydroxylase immunohistochemistry, IHC) at two time points (21 and 45 days) post MPTP model induction. We also investigate the effect of levodopa (L-DOPA) as a proof-of-principle chronic intervention against MPTP-induced changes in the retina. We show that MPTP decreases dopaminergic amacrine cell number (9%, *p* < 0.05) and that a component of the ERG that involves these cells, in particular oscillatory potential (OP) peak timing, was significantly delayed at Day 45 (7–13%, *p* < 0.01). This functional deficit was paralleled by outer plexiform layer (OPL) thinning (*p* < 0.05). L-DOPA treatment ameliorated oscillatory potential deficits (7–13%, *p* < 0.001) in MPTP animals. Our data suggest that the MPTP toxin slows the timing of inner retinal feedback circuits related to retinal dopaminergic pathways which mirrors findings from humans with PD. It also indicates that the MPTP model causes structural thinning of the outer retinal layer on OCT imaging that is not ameliorated with L-DOPA treatment. Together, these non-invasive measures serve as effective biomarkers for PD diagnosis as well as for quantifying the effect of therapy.

## Introduction

Parkinson’s disease (PD) is a multifactorial, debilitating neurodegenerative disorder of the central nervous system (CNS) that primarily affects the body’s control of voluntary movement. The pathogenesis of PD remains incompletely understood and as such there is still not a specific test that effectively diagnoses PD. Current treatment of PD usually involves the administration of dopamine agonists or related precursor drugs like levodopa (L-DOPA)^1,2^. However, these medications only treat the physical symptoms of PD, leaving underlying pathophysiological mechanisms unaddressed^3^. The retina’s accessibility provides an opportunity for gradual changes in the central nervous system to be monitored from the eye, and for abnormalities to be more readily screened for in patients at risk of certain neurological diseases, such as Parkinson’s disease. Furthermore, given that retina and brain^4^ share similar embryological origins, neurotransmitters and associated receptors, as well as blood neural barriers and transporters, retinal biomarkers may also have a role in drug development with certain ocular measures complementing existing systemic biomarkers to better ascertain drug efficacy and safety^5^.

In PD the loss in dopaminergic innervation stereotypically manifests in patients as tremor, rigidity and bradykinesia (i.e. slow movement), making motor deficits the most well characterized and recognizable signs and symptoms of the disease. However, there are a plethora of non-motor symptoms including visual dysfunctions that are becoming recognized as being important to those affected by PD^6^. Apart from being a key CNS neurotransmitter that regulates movement, dopamine plays an integral role in retinal visual processes; particularly in light adaptation^7^. Within the retina, dopamine is produced and released by a distinct subclass of neurons known as amacrine cells, which act on a family of dopaminergic receptors that are extensively located throughout the retina. Dopamine is essential to maintaining retinal neuronal survival^8^ and overall eye growth^9^ as well as facilitating a range of retinal functions, including circadian rhythms^10^ and visual sensitivity to light and spatial information. Loss of dopaminergic amacrine cells and thus reductions of retinal dopamine levels have been established in PD, which may contribute towards visual symptoms experienced by patients, such as decreased contrast sensitivity^11^. As such the characterization of retinal function and structure in a mouse model of dopaminergic modulation may afford insights into potential mechanisms through which visual symptoms manifest.

The effects of the 1-methyl-4-phenyl-1,2,3,6-tetrahydropyridine (MPTP) toxin was first observed in intoxicated people^12^ who presented with Parkinson’s-like symptoms through its effect on the dopaminergic pathway. This discovery was then translated to mice and non-human primates as a model of PD. By targeting and damaging the nigrostriatal dopamine pathway of the substantia nigra pars compacta (SNpc) in addition to the ventral tegmental area (VTA)^13,14^, MPTP initiates selective cellular apoptosis within dopaminergic neurons. Although no animal model can replicate all aspects of the human condition, the rapid and easily reproducible neurotoxic effect of MPTP on the nigrostriatal system, makes the MPTP model useful for understanding the role of SNpc neuron loss in PD as well as for evaluating therapeutic efficacy of new drugs.

Peripherally administered MPTP has neurotoxic effects in a number of animal species^15^; the most common laboratory models being primates and mice. Within both species, death of SNpc dopaminergic neurons associated with the reduction of striatal dopamine^16^ has been observed; akin to human cases of MPTP-induced PD^12^. In addition to instigating dopaminergic cell death in the SNpc and VTA, MPTP appears to also impact retinal function and dopamine levels in retinal amacrine cells. More specifically, several groups have quantified dopamine and electrophysiological changes within the retina after MPTP intraperitoneal injection in nonhuman primates^17–19^, rabbits^20,21^ and rodents^22,23^. These studies have generally found that around 7 days after systemic MPTP administration, retinal dopamine levels markedly decrease along with reduced dark-adapted electroretinogram (ERG) b-wave and oscillatory potential (OP) amplitudes^21,23^; suggesting that MPTP’s effects may be localized to bipolar cells and amacrine cells. While it has been useful to understand the acute effects of MPTP on retinal DA and function (examined 2 h—7 days post MPTP injection), few studies model the long-term pathological and compensatory changes seen in drug-induced PD^12^.

Interestingly, those chronic studies (15–20 days) of MPTP-treated non-human primates have shown that the administration of L-DOPA partially recovered losses in pattern ERG waveforms^17,24^. It remains to be seen whether MPTP treated mice also exhibit ERG changes at these longer time points, and whether any changes can be reversed with dopamine-related therapeutics.

Optical coherence tomography (OCT) as a marker for PD is gaining increasing interest, given its widespread availability and capacity for differentiating cell classes at micron resolution. In the human literature, meta-analyses show that consistent thinning in foveal thickness (which primarily contains outer retinal neurons) and the retinal nerve fibre layer have been reported ^25,26^. However, no studies have examined OCT in an MPTP model or modification with L-DOPA treatment which may inform understanding of dopamine driven changes. As such, this study assessed in vivo retinal function (electroretinography) and structure (imaging using optical coherence tomography) in MPTP- and untreated control mice at 21 and 45 days after treatment. Retinae were also harvested for ex vivo immunohistological quantification of tyrosine hydroxylase (TH) positive amacrine cell numbers. By doing so, we aim to consider whether these non-invasive retinal biomarkers are sensitive enough to detect these systemic dopaminergic modifications and their feasibility for drug screening.

## Methods

### Animals and MPTP model induction

All experimental protocols were conducted in adherence with the Howard Florey Institute Animal Experimentation Ethics Committee (Ethics approval number: 17-046-UM) and the National Health and Medical Research Council Australian Code of Practice for the care and use of animals for scientific purposes. This study was carried out in compliance with the ARRIVE guidelines^27^. A total of 97 male adult C57BL6/J mice (12–14 weeks of age) were obtained from the Animal Resource Centre (Canning Vale, WA, Australia). Mice were housed at constant room temperature (20 °C) and alternated through a 12-h diurnal light–dark cycle. Room illumination was kept to < 50 lx to minimize ocular photo-oxidative stress^28^. Food (Barastoc mouse pellets, Melbourne, VIC, Australia) and water were provided ad libitum.

The MPTP model of PD was induced by administering the MPTP neurotoxin (65 mg/kg, Sigma-Aldrich, saline vehicle) into 12–14 week old C57BL6/J male mice with 4 intraperitoneal injections (16.25 mg/kg each, delivered 2 h apart) as previously described^29^ and similar to previous literature in mice^30,31^. This dose was titrated to cause a lesion sufficient to kill approximately half of the dopaminergic neurons in the substantia nigra pars compacta (SNpc)^32^ with the expectation of also killing a significant number of dopaminergic amacrine cells^23^. Assessment endpoints were at 21 (control n = 14; MPTP-treated n = 15) and 45 (control n = 19; MPTP-treated n = 35) days post MPTP to allow chronic changes to manifest. Additionally, a group of mice was treated with L-DOPA (0.2 mg/ml with 0.2% ascorbic acid; n = 14) through their drinking water from the beginning of MPTP induction and underwent in vivo assessment 45 days after MPTP induction.

Electrophysiological recording and imaging procedures were conducted under general ketamine:xylazine anaesthesia (intraperitoneal, 80:10 mg/kg Troy Laboratory, Smithfield, NSW, Australia). A randomized set of animals from each treatment group underwent in vivo retinal assessment per experimental day. Mydriasis and corneal anesthesia were achieved using topical Mydriacyl™ (1% tropicamide, Alcon Laboratories, Frenchs Forest, NSW, Australia) and Alcaine™ (0.5% proxymetacaine, Alcon Laboratories), respectively. A heat pad was used to maintain body temperature at 37.5 ± 0.5 °C throughout.

### Electroretinography

Full-field electroretinography (ERG) was performed on all control and treatment groups at 21 and 45 days post MPTP induction. ERG assessment included dark-adapted and light-adapted responses as previously described^33,34^.

In brief, animals were dark adapted overnight to maximize retinal sensitivity^35^. Active and inactive silver chloride electrodes (A&E Metal Merchants, Sydney, NSW, Australia) were placed on the cornea and sclera, respectively. A stainless-steel ground electrode was placed in the tail (F-E2-30, Grass Telefactor, West Warwick, RI, USA). A customized Ganzfeld sphere allowed for even retinal illumination with calibrated light intensities (IL1700, International Light technologies, Peabody, MA). Data acquisition was undertaken with Scope™ software (Powerlab ADInstruments, Bella Vista, NSW, Australia) at a sampling rate of 4 kHz over a 640 ms recording window. Signals were band pass filtered (3 to 1000 Hz, − 3 dB) to minimize high frequency noise. The dark-adapted ERG protocol used light stimuli ranging from − 5.53 to 2.07 log cd s/m^2^ to elicit photoreceptoral (a-wave), bipolar cell (b-wave), amacrine cell (oscillatory potentials, OPs) and ganglion cell (scotopic threshold response, STR) mediated features of the ERG. To evaluate the light-adapted response (cone pathway), animals were adapted to a 125 cd/m^2^ background for 15 min, with responses tracked every minute (2.72 log cd s/m^2^, as shown in Supplementary Figures)^36^. Once signals had stabilized a sequence of luminous energies ranging from 0.3 to 2.72 log cd s/m^2^ elicited the cone pathway driven a-wave, b-wave and OPs.

Waveforms were analyzed offline in Excel™ (Microsoft, Redmond, WA, USA)^33^. In short, the a-wave was modelled with a P3 model^37^ which returns measures of photoreceptoral amplitude (RmP3, µV) and how efficiently light is transduced into a chemical signal (sensitivity or S, m^2^ cd s^−3^). To quantify post-receptor ON-bipolar and amacrine cell responses, the P3 model was digitally subtracted from the raw ERG waveform, to yield oscillatory potentials (OPs) and the P2 (OP-P2 complex). A discrete Fourier transform and digital filters were applied to the OP-P2 complex to isolate the bipolar cell driven P2 component (low pass filter, 46.9 Hz, − 3 dB)^38^ and OPs (band pass filter, 50 to 120 Hz, − 3 dB)^39^. Peak amplitude for P2 waveforms across all intensities was modelled using a saturating function to return measures of bipolar cell amplitude (Vmax, µV), and sensitivity to light (1/sensitivity, 1/K, log cd s/m^2^). OP waveforms were analysed for their peak amplitude (µV) and peak times (ms) following stimulus onset. Retinal ganglion cell responses were assayed with the pSTR peak amplitude (µV) and peak time (ms).

### In vivo optical coherence tomography imaging

Following ERG assessment, in vivo retinal structure was assayed with optical coherence tomography (OCT) (Spectralis^®^, Heidelberg Engineering, Heidelberg, Germany) with general anaesthesia, mydriasis and corneal hydration (Systane, Alcon Laboratories) maintained. During image acquisition, retinal volumes (7.6 × 6.3 × 1.9 mm) centered on the optic nerve head were acquired each consisting of 121 evenly spaced horizontal B-scans. Each B-scan comprised of 768 A-scans with 3.87 µm axial depth resolution, 9.8 µm lateral resolution. OCT scans were captured with an automated real-time tracking average of 6 frames, at an average speed of 85,000 A-scans per second.

The Heidelberg Eye Explorer 2 OCT reader plugin (Heidelberg Engineering) was used for analysis. All retinal layers (retinal nerve fibre layer, RNFL; ganglion cell inner plexiform layer, GCIPL; inner nuclear layer, INL; outer plexiform layer, OPL; outer nuclear layer, ONL; photoreceptor segments, PR segment) as well as total retinal thickness (TRT) were automatically segmented (Heidelberg Eye Explorer *2* software). A circular ring around the optic nerve head (Early Treatment Diabetic Retinopathy Study [ETDRS] outer ring) was employed in analysis.

### Histological and stereology assays

To determine the effect of MPTP on retinal dopaminergic amacrine cells, immunohistochemistry for tyrosine hydroxylase (TH), a key precursor for dopamine production^40^ was undertaken. TH positive amacrine cell counts were carried out on retinal flat mounts from randomly assigned animals from control and treatment groups at 21 and 45 days post MPTP injection (total of 53 eyes, n = 7–12 eyes / group).

Following in vivo assessments, mice were sacrificed, and eyes were harvested. Eyes were fixed in 4% paraformaldehyde in 0.1 M phosphate buffer solution (PBS) for 1 h at room temperature and dissected to isolate retinal tissue. Retinae were incubated in ethylenediaminetetraacetic acid (EDTA; 20 mM) for 1 h at 37 °C then blocked in PB with 5% goat serum (Sigma-Aldrich, Castle Hill, NSW, Australia, Cat no. G9023) and 0.5% Triton X-100 (ICN Biomedicals, OH, USA, Cat no. 807423). To detect dopaminergic amacrine cells, retinae were incubated with a polyclonal mouse antibody specific for the detection of TH (1:500, Chemicon^®^, Tokyo, Japan, Cat no. AB152) overnight at 4 °C on an agitator. Retinae were then washed and incubated with a secondary goat anti rabbit IgG Alexa 647 antibody (1:500; Thermo Fisher Scientific, Scoresby, VIC, Australia, Cat no. A-21244) for 1 h at room temperature and then counterstained with Hoechst nuclei stain (1:600; Roche Applied Science, Mannheim, Germany) for 10 min at room temperature. Retinae were washed, flat mounted and cover slipped with the photoreceptor side down. Retinae were imaged on the Zeiss LSM 780 confocal laser scanning microscope (Carl Zeiss, Jena, Thuringia, Germany) at 10x (0.45 NA) in 1 µm steps using 2048 × 2048-pixel resolution. A total of 4 confocal z-stacks were taken per eye, each capturing one retinal flat mount quadrant. Observers masked to treatment groups manually counted TH-positive amacrine cells using FIJI software (National Institutes of Health, Bethesda, MD, USA).

The substantia nigra pars compacta (SNpc) is composed of 95% tyrosine hydroxylase^41^, we performed a total cell count of the SNpc to determine the extent of MPTP intoxication on neuron number. The total number of neurons in the SNpc was estimated using a stereological fractionator sampling design. Brains were fixed overnight in 4% paraformaldehyde in PBS, then cryoprotected in 30% sucrose until the brains sunk, the SNpc was sectioned in a 1 in 3 series at 30 µm with a cryostat (Leica, Wetzlar, Germany) and then counter stained with neutral red. Cell counts were made at regular predetermined intervals (x = 140 µm, y = 140 µm). Systematic samples of the area occupied by the nuclei were made from a random starting point. An unbiased counting frame of known area (45 µm × 35 µm) was superimposed on the image of the tissue sections using stereology software (MBF, Stereo Investigator, Williston, VT, USA) utilizing a 63× objective lens (Leica, N.A.1.36). Experimenters were blinded to the treatments of each animal.

### Statistical analysis

All group data are expressed as group mean ± standard error of the mean (SEM) unless stated otherwise. In general, groups were compared using two-tailed unpaired Student’s *t*-tests and two-way analysis of variances (ANOVA) with Sidak correction for multiple comparisons using Prism 8 (GraphPad Software, La Jolla, CA, USA). However, in cases where datasets were found to be non-parametric following Shapiro–Wilk normality testing, Mann Whitney ranking tests were performed. For the identification of outliers, Grubbs’ test was applied.

## Results

### MPTP decreases the number of dopaminergic amacrine and substantia nigral cells

**Figure 1:**
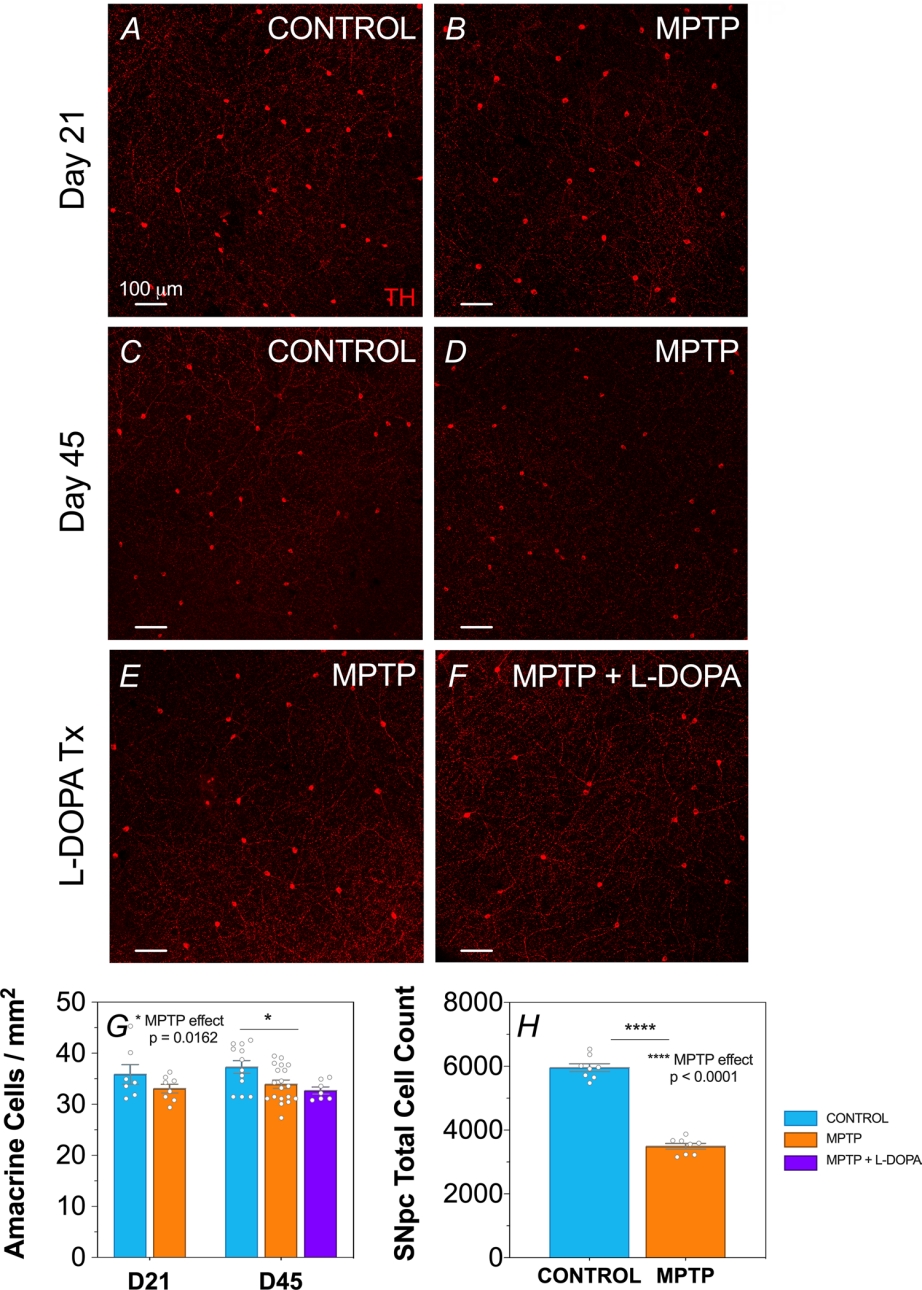
Effect of MPTP and L-DOPA on retinal and substantia nigral dopaminergic neurons. Representative retinal flatmount images of dopaminergic tyrosine hydroxylase (TH) positive amacrine cells at × 10 magnification of Day 21 (**A**,**B**), Day 45 (**C**,**D**) and Day 45 L-DOPA treatment (Tx) (**E**,**F**) cohorts. Scale bar = 100 μm. Group average (± SEM) TH-positive amacrine cell counts (**G**) of all treatment groups and total substantia nigral (SNpc) cell counts (**H**) of control vs. MPTP-treated animals at Day 45. Day 21 cohort (D21): control (n = 7, blue) vs. MPTP (n = 8, orange). Day 45 cohort (D45): control (n = 12, blue) vs. MPTP (n = 19, orange) vs. MPTP + L-DOPA (n = 7, purple).

Dopaminergic TH-positive amacrine cells assessed via immunohistochemistry were localised to the inner retina (Fig. 1A–F), between the outer and inner plexiform layers^42^. We found a significant decrease in TH-positive ACs in MPTP-treated animals compared to wildtype (WT) controls (two-way ANOVA, MPTP effect, *p* = 0.016), in particular at 45 days post MPTP induction (9% loss, post-hoc comparison, *p* = 0.046; Fig. 1G). MPTP animals that were treated with L-DOPA across the 45 days showed similar numbers of TH-positive ACs at Day 45 compared with MPTP positive controls, suggesting that L-DOPA treatment does not preserve dopaminergic ACs in this disease model. Furthermore, total cell counts of the substantia nigra performed 45 days post MPTP induction revealed a 58% reduction of neurons in MPTP-treated animals compared to WT controls (unpaired t-test, MPTP effect, p < 0.0001, Fig. 1H).

### OCT reveals outer retinal thinning in MPTP mice

**Figure 2:**
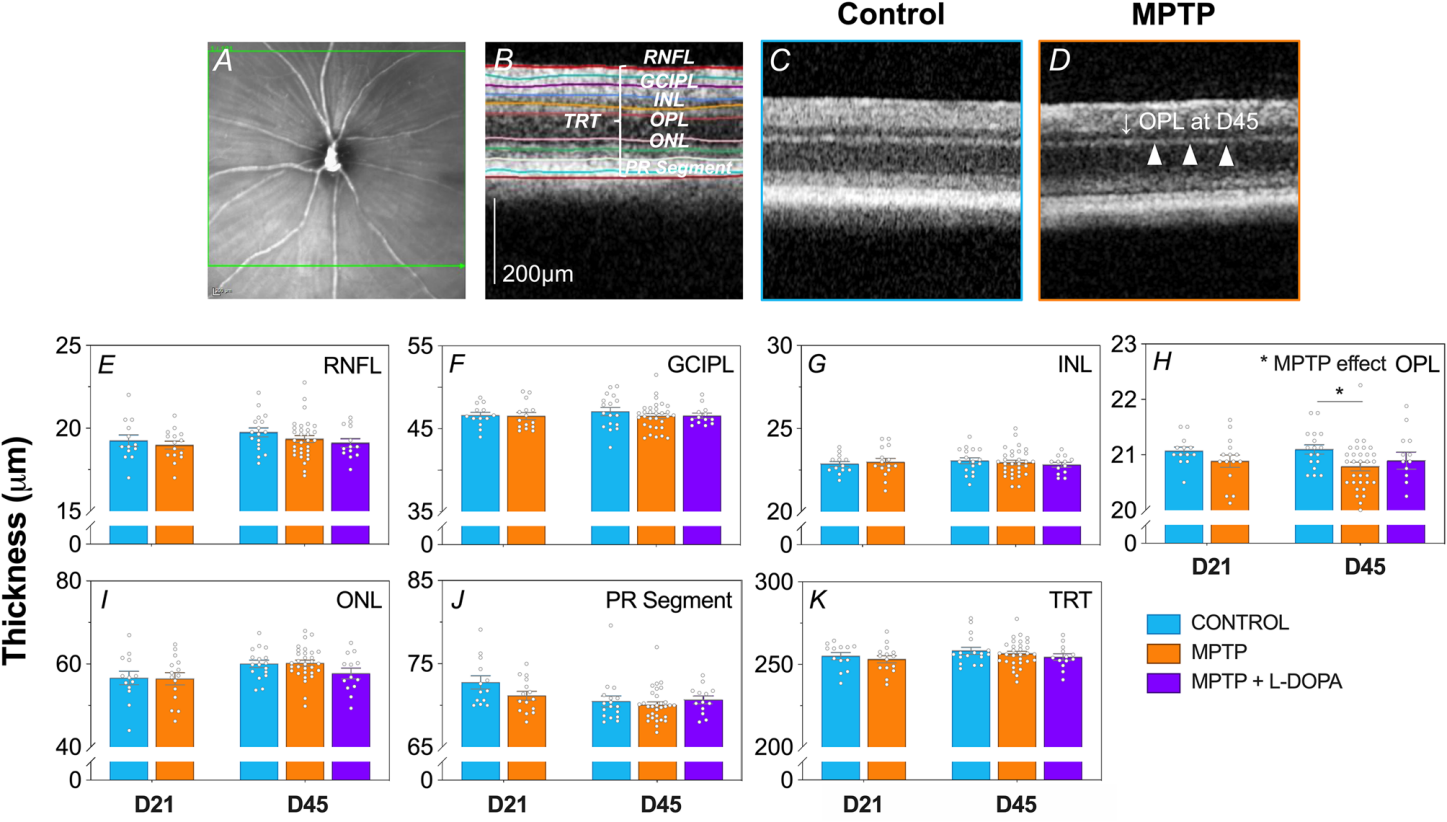
Effect of MPTP and L-DOPA on in vivo retinal structure. In vivo retinal cross-section with automated layer segmentation (**A,B**) and representative OCT images of control (**C**) MPTP treated (**D**) cohorts. Scale bar = 200 μm. Group mean (± SEM) thicknesses (μm) for retinal nerve fibre layer (RNFL; (**E**)), ganglion cell inner plexiform layer (GCIPL; (**F**)), inner nuclear layer (INL; (**G**), outer plexiform layer (OPL; (**H**)), outer nuclear layer (ONL; (**I**)), photoreceptor segments (PR segment; (**J**)) and total retina (TRT; (**K**)). Day 21 cohort (D21): control (n = 13, blue) vs. MPTP (n = 15, orange). Day 45 cohort (D45): control (n = 17, blue) vs. MPTP (n = 31, orange) vs. MPTP + L-DOPA (n = 13, purple). MPTP-treated animals had significantly thinner outer plexiform layers (OPL) compared to WT controls (two-way ANOVA, treatment effect, *p* = 0.012).

Optical coherence tomography (OCT) imaging was employed to compare in vivo retinal structure between WT control and MPTP-treated mice. Figure 2 shows representative OCT images and group averaged layer thicknesses at Day 21 and Day 45 post treatment. We found MPTP-treated animals had significantly thinner outer plexiform layers (OPL) compared to WT controls (two-way ANOVA, treatment effect, *p* = 0.012, Fig. 2H) especially at Day 45 (post-hoc comparison, *p* = 0.023). L-DOPA did not reverse this MPTP-induced thinning in the OPL (*p* = 0.496). Whilst all retinal layers trended to be thinner in MPTP-treated animals, changes to the other retinal layers were not significant (Fig. 2E–G,I–K, p = 0.079 to 0.998).

### MPTP causes inner retinal dysfunction that is ameliorated by L-DOPA treatment

**Figure 3:**
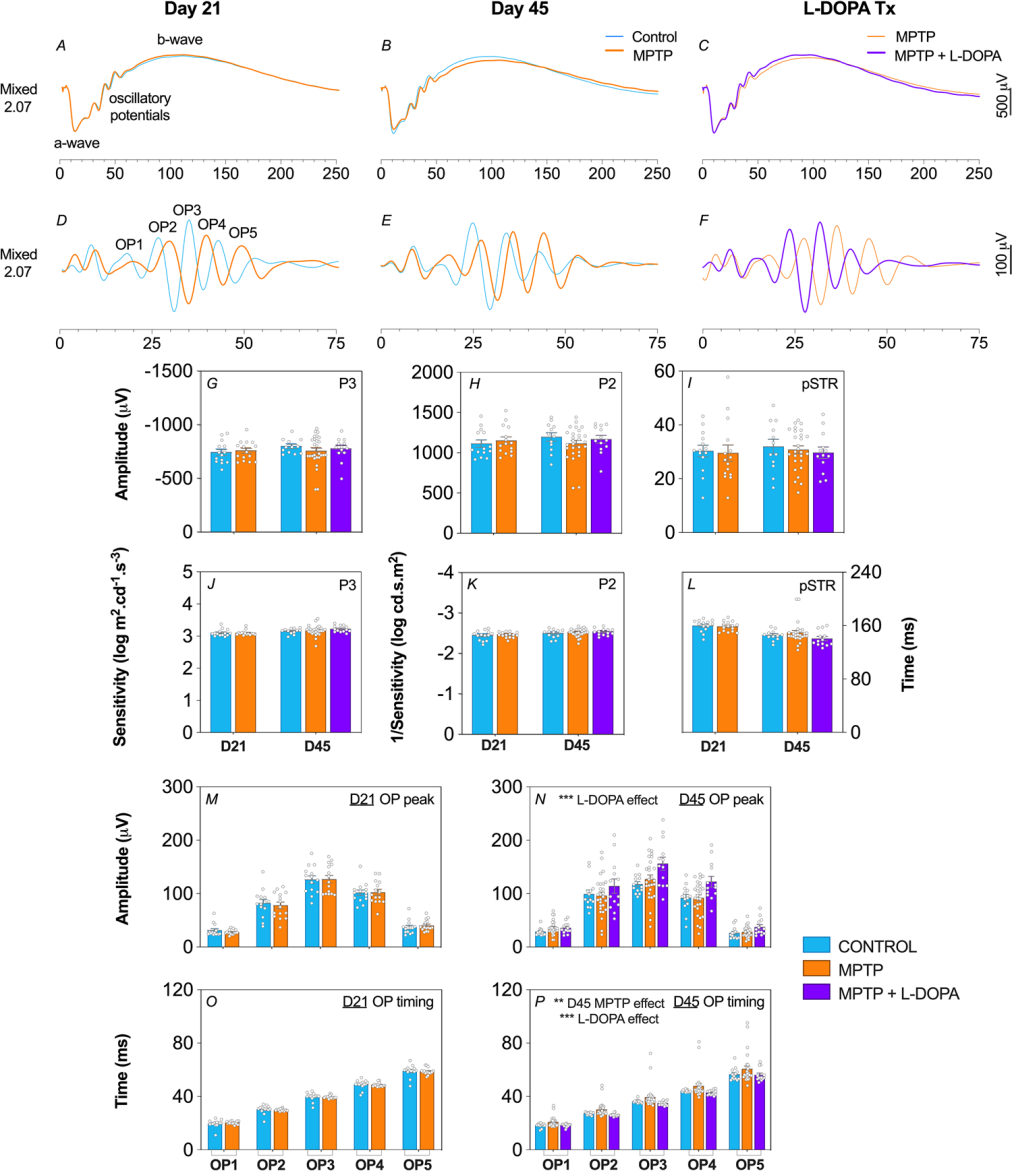
Effect of MPTP and L-DOPA on in vivo retinal function of the dark-adapted pathway. Averaged mixed rod and cone ERG waveforms (**A**–**C**) in response to a bright flash (2.07 log cd s/m^2^) eliciting photoreceptoral (P3) and ON-bipolar cell (P2) responses. Representative oscillatory potential (OP) waveforms (**D**–**F**) (2.07 log cd s/m^2^). Group average (± SEM) P3 (**G**), P2 (**H**) and positive scotopic threshold response (pSTR, (**I**)) peak amplitudes (μV). Group average (± SEM) P3 (**J**) and P2 (**K**) sensitivity. Group average (± SEM) pSTR (**L**) timing (ms). Group average (± SEM) OP1 to OP5 amplitude (**M**,**N**) and timing (**O**,**P**) of Day 21 and Day 45 animals, respectively. Day 21 cohort (D21): control (n = 14, blue) vs. MPTP (n = 15, orange). Day 45 cohort (D45): control (n = 12, blue) vs. MPTP (n = 27, orange) vs. MPTP + L-DOPA (n = 13, purple).

**Figure 4:**
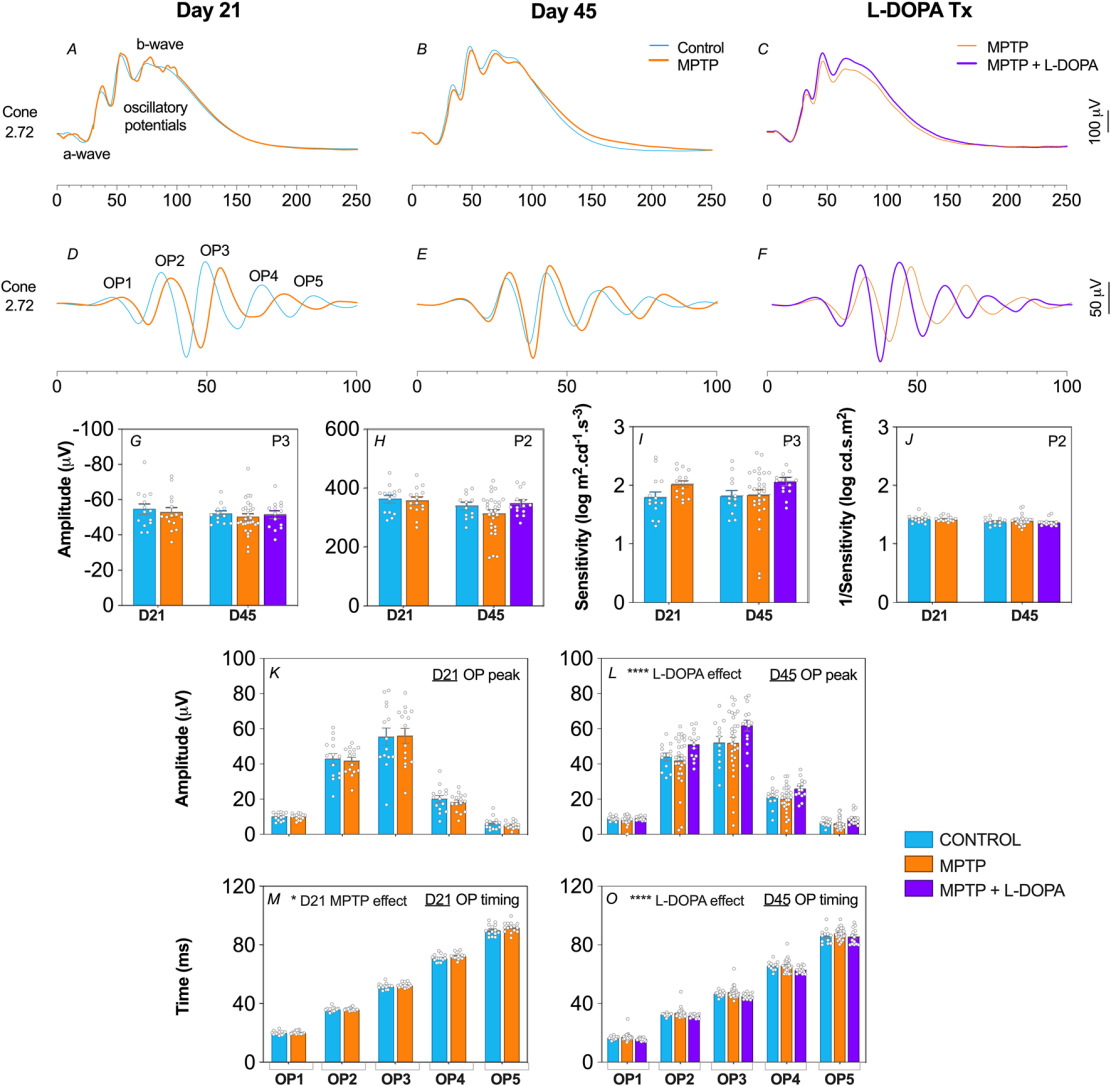
Effect of MPTP and L-DOPA on in vivo function of the light-adapted pathway. Averaged cone ERG waveforms (**A**–**C**) in response to a bright flash (2.72 log cd s/m^2^) inducing photopic a-wave and ON-bipolar cell b-wave. Representative oscillatory potential (OP) waveforms (**D**–**F**) (2.72 log cd s/m^2^). Group average (± SEM) P3 (**G**) and P2 (**H**) peak amplitudes (μV). Group average (± SEM) P3 (**I**) and P2 (**J**) sensitivity (log cd s/m^2^). Group average (± SEM) OP1 to OP5 amplitude (**K**–**L**) and timing (**M**–**O**) of Day 21 and Day 45 animals, respectively. Day 21 cohort (D21): control (n = 14, blue) vs. MPTP (n = 15, orange). Day 45 cohort (D45): control (n = 12, blue) vs. MPTP (n = 29, orange) vs. MPTP + L-DOPA (n = 14, purple).

Full field electroretinography (ERG) was used to compare retinal function. Dark-adapted (Fig. 3, including both rod and cone) and light adapted (Fig. 4, cone only) responses were measured. At the brightest light level (2.07 log cd s/m^2^), the ERG waveform comprises of a large negative photoreceptoral a-wave followed by a large bipolar cell b-wave (dark-adapted in Fig. 3A–C and light-adapted in Fig. 4A–C) on which the high frequency amacrine cell mediated oscillatory potentials (OPs) are evident. The corresponding dark-adapted and light-adapted OP waveforms isolated after digital filtering are shown in Figs. 3D–F and 4D–4F, respectively.

At 21 days post MPTP model induction, ERG waveforms were largely similar between control and MPTP-treated mice. All dark-adapted and light-adapted ERG parameters were unaffected by MPTP apart from a delay in the timing of light-adapted OPs (two-way ANOVA, MPTP effect, *p* = 0.017, Fig. 4M).

More severe effects were evident at 45 days after MPTP injection as shown by the average ERG waveforms in Figs. 3B,E and 4B,E. In particular, there were significantly delayed dark-adapted OP peak times (two-way ANOVA, treatment effect, *p* = 0.002, Fig. 3P) with a trend for delayed light-adapted OP peak times that did not reach significance (*p* = 0.106). No other ERG parameters were found to be significantly affected.

L-DOPA administration to MPTP mice significantly improved both dark-adapted (OP peak amplitude, two-way ANOVA, L-DOPA effect, 19–33% increase, *p* = 0.0002, Fig. 3N; OP timing, two-way ANOVA, L-DOPA effect, 7–13% faster, *p* = 0.0001, Fig. 3P) and light-adapted (OP peak amplitude, two-way ANOVA, L-DOPA effect, 13–48% increase, *p* < 0.0001, Fig. 4L; OP timing, two-way ANOVA, L-DOPA effect, 4–8% faster, *p* < 0.0001, Fig. 4O) amacrine cell derived OP parameters. Importantly, we observed a therapeutic reversal effect of MPTP –induced OP timing delay when compared to WT controls (Figs. 3P, 4O).

## Discussion

Whilst MPTP’s neurotoxic effects on the brain and behaviour has been well characterized^43,44^, here we show that MPTP also produces robust changes in the rodent eye. In particular, using a comprehensive suite of full field ERG measures we show that OP peak times were affected and were significantly delayed in MPTP-treated cohorts, particularly at 45 days after MPTP administration. These functional deficits coincided with thinning in the outer plexiform layer (OPL) and a decrease in dopaminergic (DA-ergic) tyrosine hydroxylase (TH) positive amacrine cells (AC) in MPTP-treated animals 45 days post induction. Furthermore, we show that L-DOPA could ameliorate OP delays, without improving TH-AC cell numbers or changing outer retinal thickness.

### MPTP moderately affects retinal structure in mouse eyes

To the best of our knowledge, no previous study has attempted to use OCT to quantify retinal thicknesses in MPTP-treated mice. Our data are the first to show in an experimental model specific OPL thinning, especially at Day 45. This is broadly in line with meta-analyses of human PD patients^25,26^ and MPTP treated monkeys^45^ showing consistent thinning of the central foveal region where outer retinal cells predominate. In particular, the foveal area includes the OPL, Henle fibres (which appear continuous with the ONL), outer nuclear layer and photoreceptor segments. Given rodents are afoveate mammals^46^, we were unable to directly compare against overall foveal thinning found in primates. Why the outer retina is thinner in MPTP mice is unclear, however it is of interest that chronic L-DOPA treatment does not ameliorate this change (Fig. 2H) suggesting dopamine supplementation cannot prevent the MPTP-induced reduction in neuronal synaptic volume.

In contrast, meta-analyses across three studies^47–49^ that have segmented the OPL do not show a consistent effect^26^. Whether this variability in the human literature is due to the difficulty in accurately segmenting the outer plexiform layer of a human fovea/macula^50,51^ or the overlay of other potential PD pathological mechanisms (i.e. a-syn deposition^52^ and dopamine depletion) requires further investigation. OCT findings in human PD patients^25,26^ and monkeys treated with MPTP^45^ also reveal thinning of the RNFL. Our data indicate a trend towards thinner RNFL in MPTP mice however this was not significant (Fig. 2A; *p* = 0.238).

At the cellular level, we detected a decrease in TH-positive amacrine cells at Day 45 (*p* = 0.046, Fig. 1G). Our level of amacrine cell loss is milder than of Takatsuna et al.^23^ who reported a 50% loss of TH-positive ACs in MPTP-treated mice as early as 10 days after MPTP injection that persists for at least until 50 days post intoxication. The severity of amacrine cell loss induced by Takatsuna et al.^23^ is likely to be related to their higher MPTP dose of 150 mg/kg, as compared with the 65 mg/kg used in the current study and the transient loss of TH phenotype at the shorter timepoint. This is consistent with the observation that dopaminergic amacrine cell loss in the mouse retina is highly dependent on MPTP dose and dosing protocols^53^. In saying that, we did observe a ~ 58% reduction of neurons in SNpc in MPTP-treated mice compared to WT controls in our study (*p* < 0.0001, Fig. 1H), confirming that our MPTP lesioning was adequate.

In summary, in a rodent model of cortical and retinal dopaminergic cell loss, our data shows a thinner synaptic layer between photoreceptors and bipolar cells, and this tissue volume does not return by supplementing with L-DOPA. This finding informs which OCT changes in human PD patients may be driven by dopaminergic mechanisms.

### MPTP causes dopaminergic amacrine cell dysfunction

Given that the majority of MPTP retinal studies in the past have only looked at the dark-adapted ERG, we elected to also interrogate the light-adapted ERG in our MPTP murine model of PD since dopamine is known to play a key role in photopic responses^7,54^. On the whole, our ERG data suggest that MPTP pathology with the current dosing regimen specifically alters the amacrine cell driven OPs. In particular, we discovered OP timing to be the most sensitive ERG parameter to MPTP treatment and L-DOPA treatment, consistent with its effects on dopaminergic amacrine cells^22^.

The dopaminergic cell class present within the retina is the A18 amacrine cell which stains positively for TH, a key precursor for dopamine biosynthesis^55^. Although it is known that OP generation reflects inner retinal inhibitory activity involving amacrine cells, it is not clear whether specific subclasses of ACs correspond to earlier or later OPs^56^. Generally, we found that dark-adapted OPs were affected by both disease and therapeutic manipulations. We found a subtle yet significant timing delay in light-adapted OPs in the MPTP-treated cohort at Day 21 (*p* = 0.017, Fig. 4M) and then we observed clear delays in dark-adapted OPs at Day 45 (*p* = 0.002, Fig. 3P).

Although others have reported OP attenuation and delays with MPTP, our study is the first to show that OP timing changes can occur in the absence of deficits in other ERG components. In addition to OP changes, reductions in photoreceptor a-wave and bipolar cell b-wave amplitudes have also been observed in MPTP-treated rodents^23^, rabbits^20,21^ and monkeys^18,57^. Thus, we believe a mild loss of dopaminergic amacrine cells leads to a small delay in the OPs, and it may be that greater amacrine cell loss with higher MPTP doses is needed to attenuate OP amplitudes and other ERG components. These findings support the idea that the ERG has clinical utility for neurodegenerative disorders within which changes to central dopamine levels occur^58^.

### Functional recovery of dopaminergic findings with L-DOPA treatment

Another key outcome of this study was that we were able to assess L-DOPA’s therapeutic effects on retinal function and structure when administered at the same time as MPTP treatment. Few animal studies have evaluated the effects of L-DOPA on retinal function, however dopamine is known to alter the receptive fields of multiple cell classes within the retina^59^. Compared to MPTP positive controls, we observed that both dark- and light-adapted OP peak amplitude (Figs. 3N, 4L) and timing (Figs. 3P, 4O) were respectively increased and faster in MPTP animals treated with L-DOPA. Similar findings have been observed in human patients^60,61^ after L-DOPA treatment. Interestingly, L-DOPA did not affect retinal thickness nor alter TH-positive amacrine cell numbers when compared to MPTP controls. As such L-DOPA does not have neuroprotective effects on retinal structure in the MPTP mouse model of PD, but appears to improve function of remaining neurons. Further studies of TH-AC dendritic morphology and synapses would be useful in understanding retinal functional deficits and L-DOPA induced amelioration. In models of the L-DOPA nigrostriatal system, neuronal compensatory changes appear to be important in sustaining dopamine function and may contribute to the symptoms of PD^62^.

Finally, although MPTP is invaluable in recapitulating some aspects of the pathophysiological changes seen in PD^63^, MPTP does not perfectly model PD. As such, care should be taken when drawing parallels between the findings in this study and the pathological anatomical changes observed in patients who have been living with PD for decades.

## Conclusions

In summary, we show that MPTP induces reproducible and robust changes in the retina, with specific changes to ERG oscillatory potentials (OPs) that is associated with a mild loss of dopaminergic amacrine cells and thinning of the outer plexiform layer. The specificity of the functional ocular biomarker was exemplified by our finding that L-DOPA treatment ameliorated delays in the OPs. Importantly, these findings reflect the retinal changes in function and structure reported in the existing human PD literature. Thus, these findings deepen our understanding of dopamine driven retinal changes and support the utility of the MPTP murine model of PD as a high-throughput means through which novel dopaminergic therapeutic compounds can be preclinically screened, through the use of retinal biomarkers, to quantitatively assess their drug efficacy and safety.

## Supplementary Information

Supplementary Figures.

## Data Availability

The authors confirm that we will adhere to the journals data availability policies including making materials, data and associated protocols promptly available to readers without undue qualifications in material transfer agreements.
