# Supplementary material for: Characterization of retinal function and structure in the MPTP murine model of Parkinson’s disease
Source: Sci Rep. 2022 May 9;12:7610. doi: 10.1038/s41598-022-11495-z (PMC9085791; doi:10.1038/s41598-022-11495-z)
Supplement: Supplementary file 1 — Supplementary Figures. [file 41598_2022_11495_MOESM1_ESM.pdf]

Supplementary Information: Characterization of Retinal Function & Structure in the MPTP Murine Model of Parkinson's Disease

Katie KN Tran[1], *Vickie HY Wong*[1], *Jeremiah KH Lim*[1*], *Ali Shahandeh*[1], *Anh Hoang* [1], *David I. Finkelstein*[2], *Bang V. Bui*[1], *Christine T.O. Nguyen*[1#]

[1] Ocular Biomarkers Laboratory, Department of Optometry and Vision Sciences, University of Melbourne, Parkville, 3010, Victoria, Australia.

[2] Parkinson's Disease Laboratory, The Florey Institute of Neuroscience and Mental Health, The University of Melbourne, Parkville, 3010, Victoria, Australia.

[*]Current address: Caring Futures Institute, College of Nursing and Health Sciences, Flinders University, Bedford Park, 5042, South Australia, Australia

#Correspondence: Christine TO Nguyen: Department of Optometry and Vision Sciences, University of Melbourne, Parkville, 3010, Victoria, Australia.

Ph: +61 3 9035 3186

E: christine.nguyen@unimelb.edu.au

Supplementary Figures

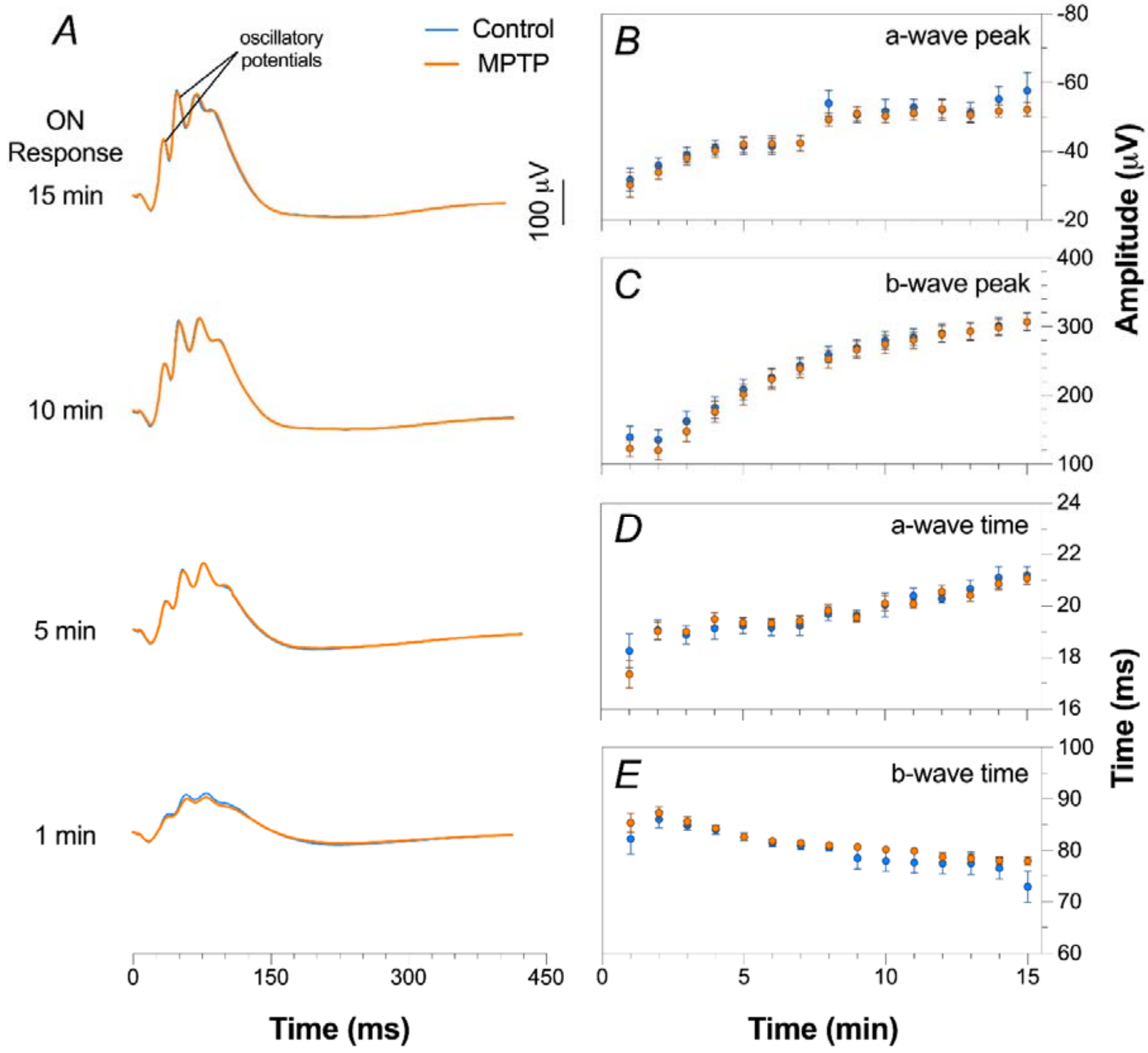

Supplementary figure 1: Day 21 photopic ERG waveform growth summary. (**A**) Representative ERG cone waveforms for control (n = 14, blue) and MPTP (n = 15, orange) cohorts, gradually growing to full saturation at 15 min. (**B & C**) a-wave and b-wave amplitude growth respectively over the 15 min light adaptation period. (**D & E**) a-wave and b-wave peak times respectively over the 15 min light adaptation period.

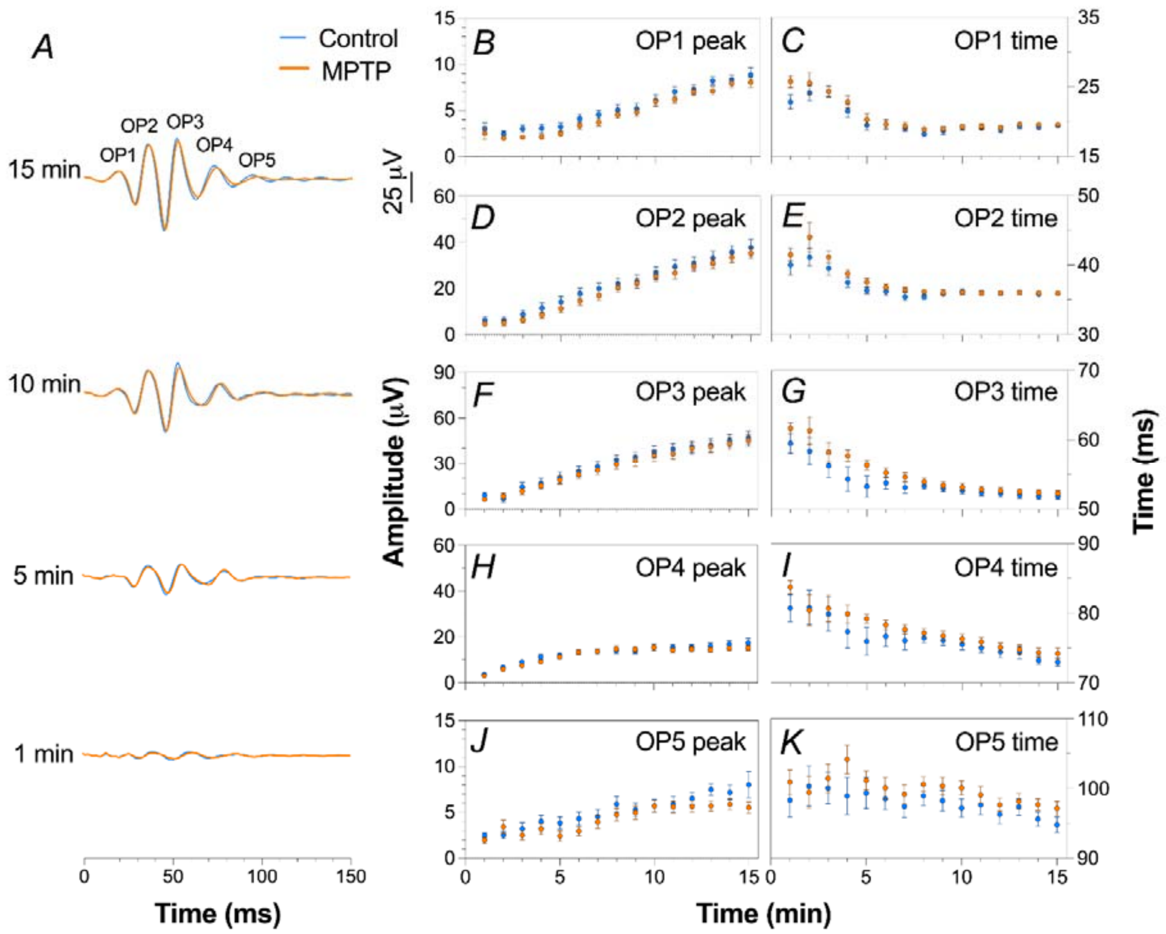

Supplementary figure 2: Day 21 photopic OP waveform growth summary. (**A**) Representative oscillatory potentials (OPs) for control (n = 14, blue) and MPTP (n = 15, orange) cohorts, growing to full saturation at 15 min. (**B & C**) OP1 peak and timing growth. (**D & E**) OP2 peak and timing growth. (**F & G**) OP3 peak and timing growth. (**H & I**) OP4 peak and timing growth. (**J & K**) OP5 peak and timing growth.

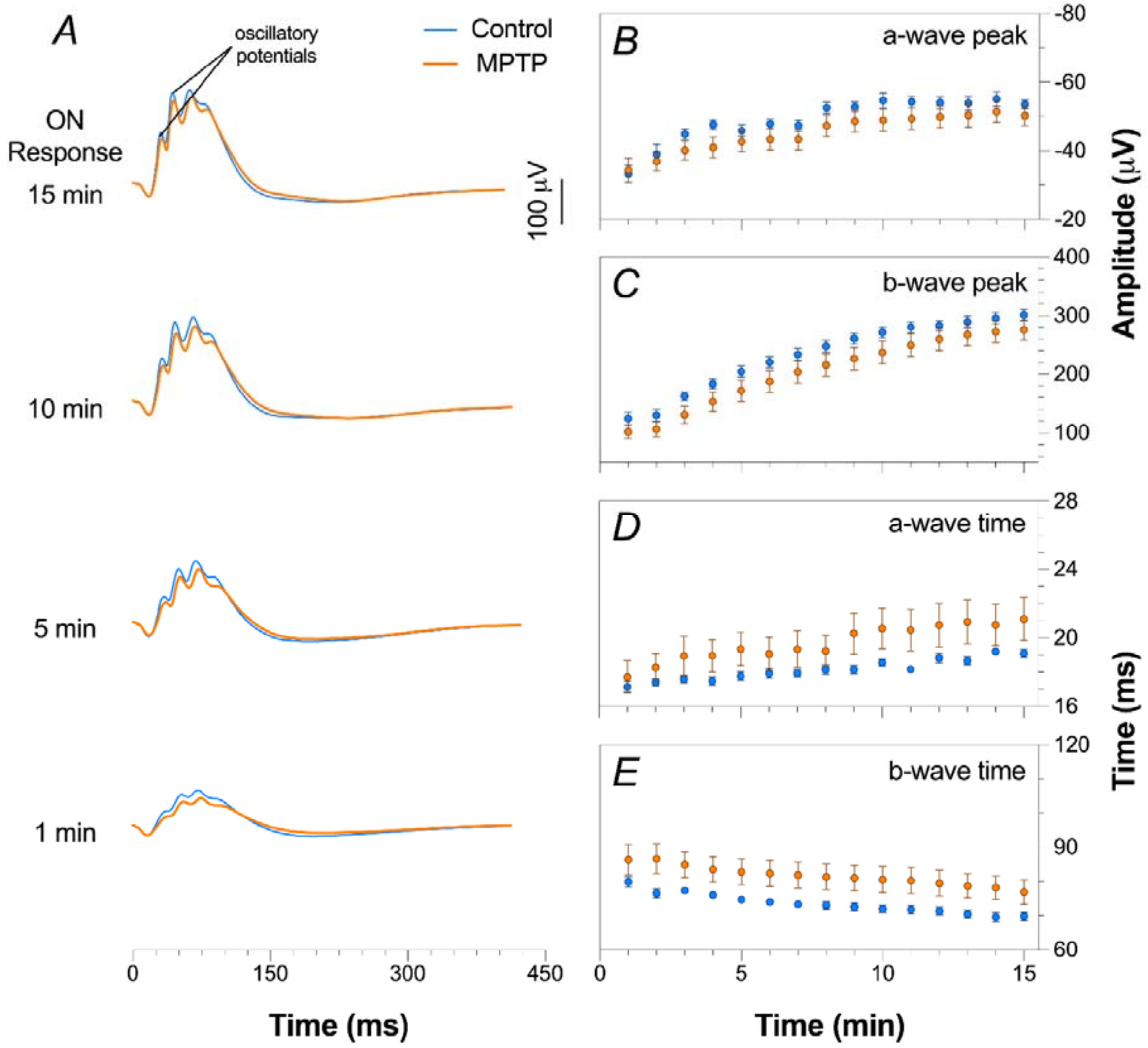

Supplementary figure 3: Day 45 photopic ERG waveform growth summary. (**A**) Representative ERG cone waveforms for control (n = 12, blue) and MPTP (n = 15, orange) cohorts, gradually growing to full saturation at 15 min. (**B & C**) a-wave and b-wave amplitude growth respectively over the 15 min light adaptation period. (**D & E**) a-wave and b-wave peak times respectively over the 15 min light adaptation period.

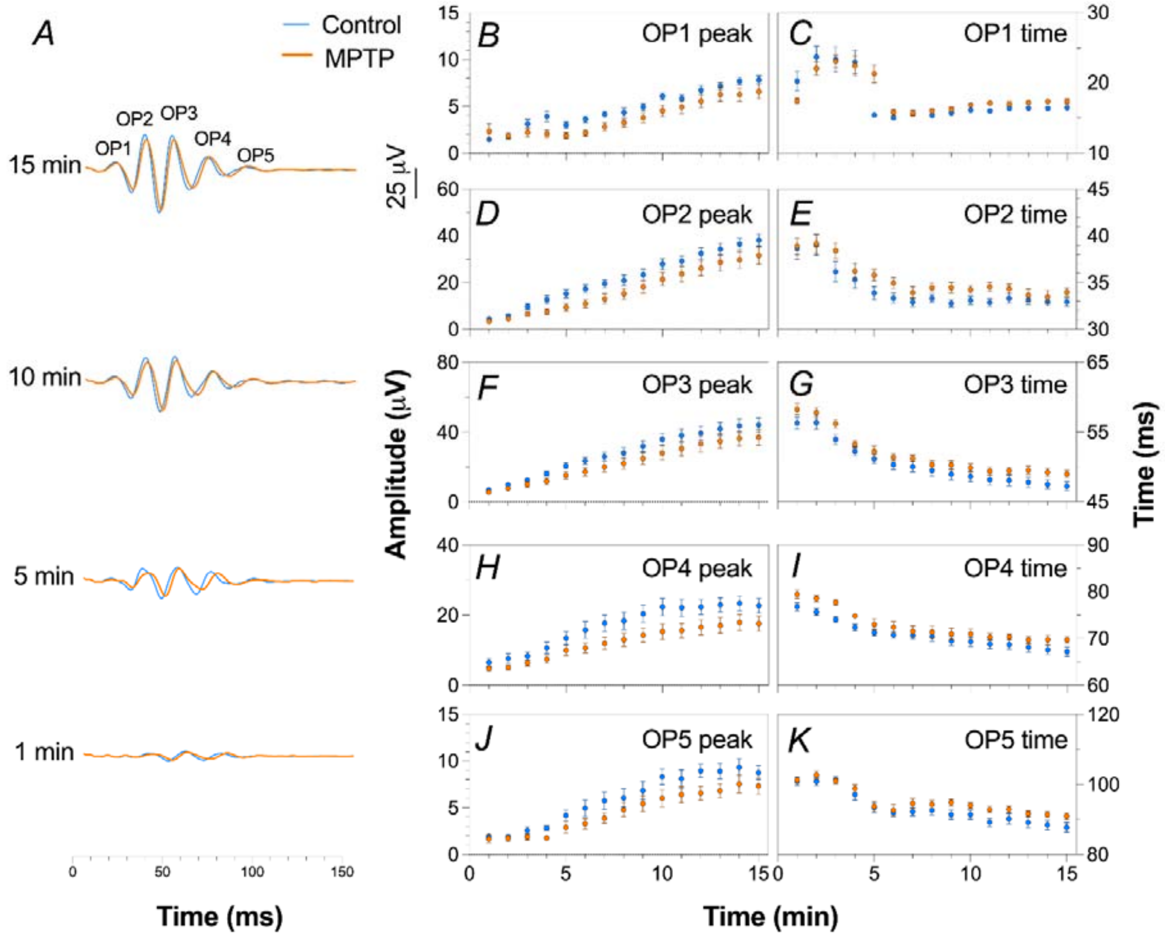

Supplementary figure 4: Day 45 photopic OP waveform growth summary. (**A**) Representative oscillatory potentials (OPs) for control (n = 12, blue) and MPTP (n = 15, orange) cohorts, growing to full saturation at 15 min. (**B & C**) OP1 peak and timing growth. (**D & E**) OP2 peak and timing growth. (**F & G**) OP3 peak and timing growth. (H & I) OP4 peak and timing growth. (**J & K**) OP5 peak and timing growth.

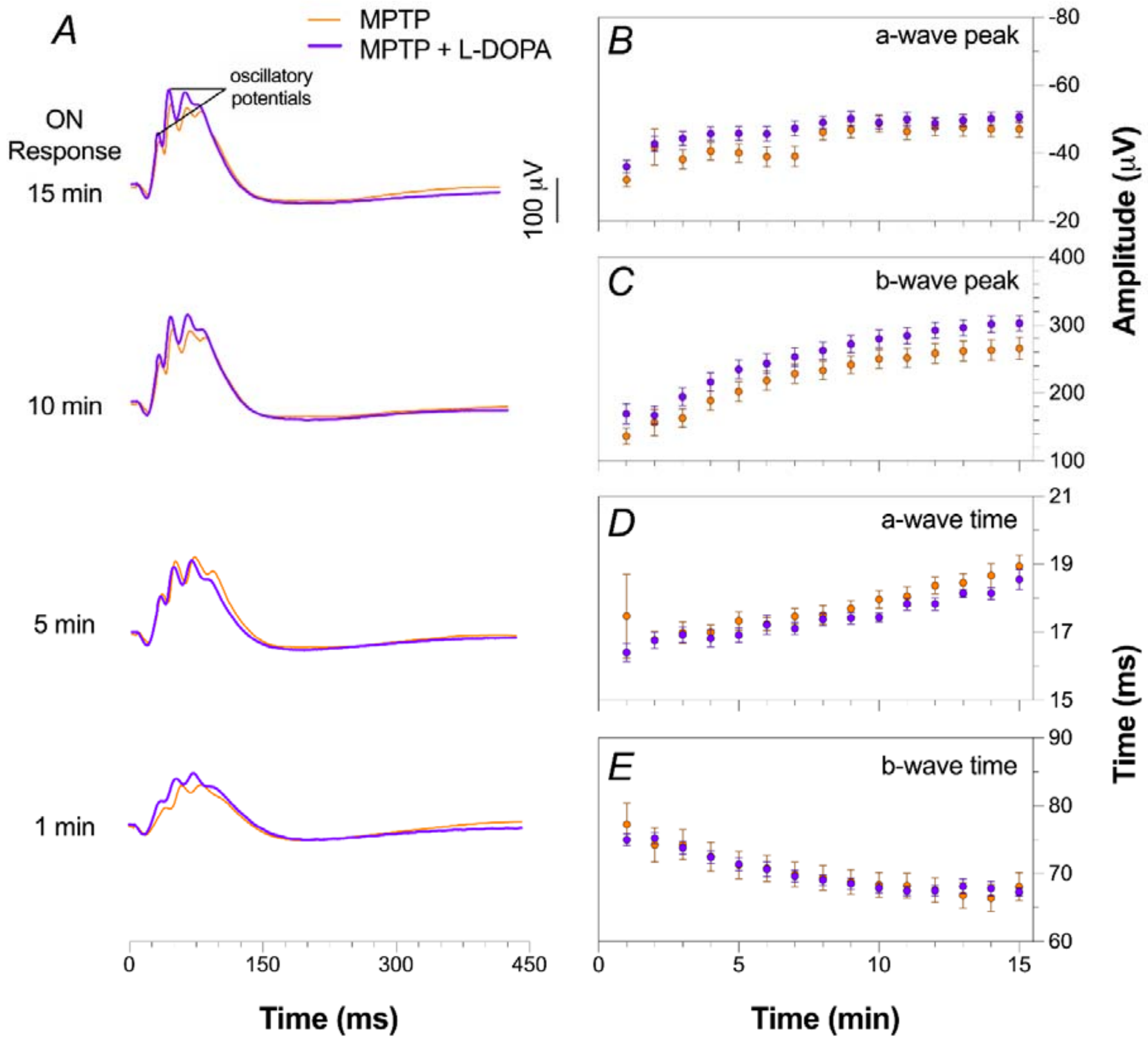

Supplementary figure 5: Day 45 L-DOPA photopic ERG waveform growth summary. (**A**) Representative ERG cone waveforms for MPTP positive control (n = 14, orange) and MPTP + L-DOPA (n = 12, purple) cohorts, gradually growing to full saturation at 15 min. (**B & C**) a-wave and b-wave amplitude growth respectively over the 15 min light adaptation period. (**D & E**) a-wave and b-wave peak times respectively over the 15 min light adaptation period.

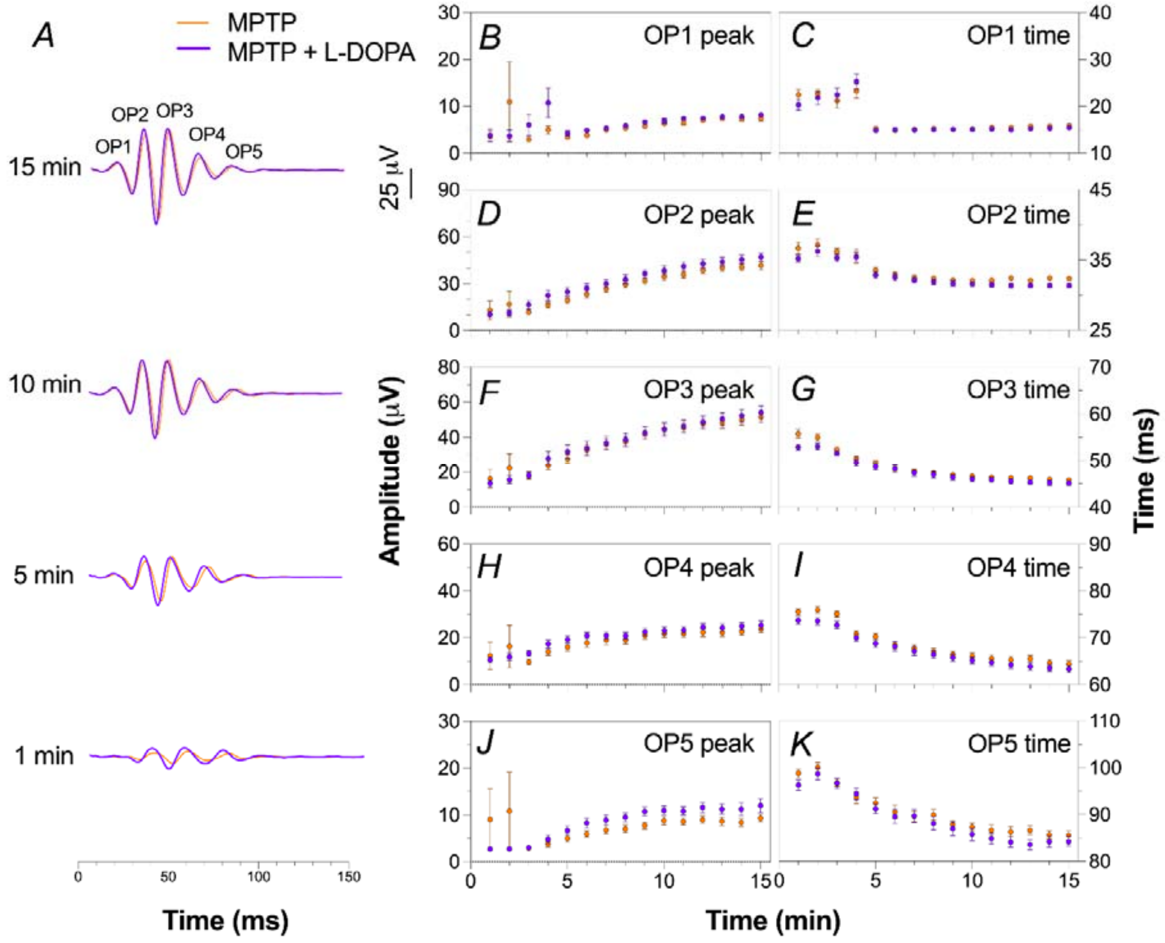

Supplementary figure 6: Day 45 L-DOPA photopic OP waveform growth summary. (**A**) Representative oscillatory potentials (OPs) for MPTP positive control (n = 14, orange) and MPTP + L-DOPA (n = 12, purple) cohorts, growing to full saturation at 15 min. (**B & C**) OP1 peak and timing growth. (**D & E**) OP2 peak and timing growth. (**F & G**) OP3 peak and timing growth. (**H & I**) OP4 peak and timing growth. (J & K) OP5 peak and timing growth.
